# Testing the Use of “Clinical Checks” With the International Trauma Questionnaire to Measure PTSD and Complex PTSD

**DOI:** 10.1111/acps.13799

**Published:** 2025-03-23

**Authors:** Mark Shevlin, Philip Hyland, Chris R. Brewin, Marylene Cloitre, Thanos Karatzias, Enya Redican

**Affiliations:** ^1^ School of Psychology Ulster University UK; ^2^ Department of Psychology Maynooth University Ireland; ^3^ Division of Psychology and Language Sciences University College London London UK; ^4^ Silver School of Social Work New York University New York New York USA; ^5^ School of Health & Social Care Edinburgh Napier University Edinburgh UK

**Keywords:** CPTSD, diagnosis, ICD‐11, PTSD

## Abstract

**Background:**

The International Trauma Questionnaire (ITQ) is the most widely used measure of ICD‐11 Posttraumatic Stress Disorder (PTSD) and Complex PTSD (CPTSD). This self‐report scale has been used to estimate prevalence rates of these disorders in general populations and clinical samples, but concerns abound that prevalence estimates derived from self‐report measures are too high. To address this concern, we previously introduced the concept of adding “clinical checks” to self‐report measures to ensure initial responses reflected the intended clinical meaning of the scale item. Here we provide a rationale for adding clinical checks to the ITQ, describe the process of developing them, and demonstrate their effect at the symptom, cluster, and disorder levels in a general population sample.

**Methods:**

A team of researchers and clinicians, including those who developed the ITQ, developed clinical checks for all ITQ items. These were tested using data from a non‐probability quota‐based representative sample of adults from the United Kingdom (*N* = 975).

**Results:**

Use of clinical checks led to decreases in symptom endorsements ranging from 18.0% to 43.9%, and symptom cluster requirements from 19.1% to 35.9%. Disorder prevalence estimates without the clinical checks were 5.4% for PTSD and 9.5% for CPTSD. With the clinical checks, prevalence estimates dropped to 3.8% for PTSD (relative decrease = 29.6%) and 4.9% for CPTSD (relative decrease = 48.4%).

**Conclusion:**

Clinical checks can be easily embedded into the ITQ and have a significant effect on prevalence estimates. We contextualize these results in relation to existing literature on population prevalence estimates derived from clinical interviews and discrepancies between clinical interviews and self‐report measures.

**Summary**

- Significant outcomes
  ○ Clinical checks reduce ITQ symptom endorsements and overall PTSD/CPTSD prevalence rates.
  ○ The ITQ with clinical checks provides an alternative approach to assess PTSD and CPTSD, combining key strengths of self‐report measures and clinical interviews.
- Limitations
  ○ The non‐probability sampling method limits generalizability.
  ○ Concordance between interview assessments and the ITQ with clinical checks was not examined.
  ○ Information is lacking on individuals who failed clinical checks and the reasons why.

## 1 Introduction

Self‐report scales are a quick and inexpensive way to measure the symptoms of a psychological disorder and ascertain if diagnostic requirements are met. This form of assessment is ubiquitous [1], but the development of the items that comprise such scales is not straightforward [2]. Items are designed to describe symptoms, and symptoms of psychological disorders can reflect affective states, cognitive processes, or overt behaviors. Symptoms of psychological disorders do not always differ in an obvious qualitative way from normal feelings, thoughts, and behaviors; often they differ quantitatively. For example, it is perfectly normal and adaptive to worry, but worry can become an indicator of psychopathology when its pervasiveness, intensity, and frequency cause substantial distress and impairment. Therefore, scale items must be capable of describing psychological phenomena in a way that distinguishes symptoms from normal experiences. This is not an easy task when the typical scale item is intended to be a short, simply worded phrase or sentence that is easily understood by all.

There is evidence that self‐report scale items may sometimes fail to capture the important clinical essence of symptoms [3]. Compared to clinical interviews, self‐report scales often produce higher rates of endorsement for specific symptoms and disorders [4]. This may be due to scale items being unable to adequately convey the intended meaning, intensity, or duration of an experience that makes it symptomatic. Taking the example of worry again, the ICD‐11 [5] description of generalized anxiety disorder describes worry in the following terms: “…*excessive worry focused on multiple everyday events…[that] persist for at least several months, for more days than not*”. Therefore, any item developed to measure this symptom needs to capture the nature of the experience (worry about multiple everyday events), its intensity (excessive worry), and its duration (more days than not, persisting for months). Sometimes all of these important aspects cannot be captured by a single item in a self‐report questionnaire, increasing the likelihood of “false‐positive” endorsements. The risk of these types of false positives can be reduced by using follow‐up checks or clarifications about the clinical relevance of the response; traditionally these “clinical checks” have only been embedded within clinician‐administered interviews. However, interviews are time consuming, costly, and impractical in many research and clinical settings.

Shevlin et al. [6] introduced the concept of “clinical checks” within self‐report questionnaires. Clinical checks are follow‐up questions to scale items that check whether respondents understood the intended clinical meaning, intensity, or duration elements. Clinical checks were intended to increase confidence in prevalence estimates derived from self‐report measures by reducing the likelihood of false positive cases. In a “proof of principle” study with the International Grief Questionnaire [7]—a self‐report measure of ICD‐11 Prolonged Grief Disorder (PGD)—Shevlin et al. reported that clinical checks led to decreases in symptom endorsements ranging from 10.8% to 53.6%, and there was a 24.8% relative reduction in the proportion of people who screened positive for PGD (from 13.6% to 10.2%).

It was proposed that clinical checks could be embedded in other measures, and so this study was planned to develop and test the use of clinical checks in the International Trauma Questionnaire (ITQ) [8], a widely used self‐report measure of ICD‐11 posttraumatic stress disorder (PTSD) and complex PTSD (CPTSD). Consistent with Shevlin et al.'s [6] findings, it was hypothesized that the application of the clinical checks would lead to statistically significant reductions in individual symptom endorsements, symptom cluster endorsements, and overall prevalence estimates of PTSD and CPTSD.

## 2 Aims of the Study

The study sought to evaluate the implementation of clinical checks in the ITQ using data from a non‐probability quota‐based representative sample of adults in the United Kingdom.

## 3 Materials and Methods

### 3.1 Procedures and Participants

An a priori power analysis was performed to determine the appropriate sample size needed to detect a disorder with a 5% population prevalence rate (assumed to be the most conservative estimate of the prevalence of PTSD in the United Kingdom [UK] population [9]), with a 99% confidence level and a 2% margin of error. This resulted in a necessary sample size of 782. Participants (*N* = 975) were gathered by Qualtrics Panel Services from March 1–27, 2024. Qualtrics partners with dozens of UK‐based research panel providers to recruit participants from a large pool of potential participants, and prior research shows that these samples are highly representative of target populations [10, 11], including the general adult population of the UK [12]. Quota sampling was used to construct a sample that was representative of the general adult population of the UK in terms of sex, age, nationality (i.e., England, Scotland, Wales, and Northern Ireland), and income level. Potential participants were contacted by Qualtrics via email or in‐app notification. Participants provided consent to participate, and ethical approval was granted by the Social Research Ethics Committee at Maynooth University (ref: SRESC‐2023‐37,628). Multiple attention checks were used throughout the survey, and Qualtrics employs different methods to ensure valid responses such as the use of CAPTCHA technology to prevent bot access and removal of responses from duplicate IP addresses, those with suspicious patterns of responding, and those deemed to have completed the survey too quickly. All participants passed all of these attention and quality control checks. The sociodemographic details for the sample are presented in Table 1.

**TABLE 1:** Sociodemographic details for the sample (*N* = 975).

	*n*	%
Sex		
Male	473	48.5
Female	502	51.5
Age		
18–24	114	11.7
25–34	191	19.6
35–44	182	18.7
45–54	164	16.8
55–64	133	13.6
65+	191	19.6
Born in UK	871	89.3
Region of UK		
England	842	86.4
Wales	48	4.9
Scotland	66	6.8
Northern Ireland	19	1.9
Annual income		
Less than £20,000	263	27.0
£20,000–£39,999	336	34.5
£40,000–£59,999	190	19.5
£60,000–£79,999	101	10.4
£80,000–£99,999	50	5.1
More than £100,000	35	3.6
Highest education		
No qualification	39	4.0
O‐level/GCSE or similar	247	25.3
A‐level or similar	278	28.5
Undergraduate degree	282	28.9
Postgraduate degree	129	13.2
Employment status		
Full‐time employed	447	45.8
Part‐time employed	170	17.4
Unemployed, seeking work	51	5.2
Unemployed, not seeking work	45	4.6
Not working due to disability	49	5.0
Student	30	3.1
Retired	183	18.8
Relationship status		
In a committed relationship	694	71.2
Not in a committed relationship	281	28.8
Children		
0	345	35.4
1	228	23.4
2	248	25.4
3	109	11.2
4 or more	45	4.6

### 3.2 Measures

#### 3.2.1 Trauma Exposure

The International Trauma Exposure Measure [ITEM] [13] assesses lifetime exposure to 21 potentially traumatic events in a manner consistent with the ICD‐11's definition of trauma (i.e., any event that is extremely threatening or horrific). The ITEM contains descriptions of events that are traditionally regarded as traumatic (e.g., physical assault, sexual assault, exposure to war) as well as events that are regarded as traumatic by the ICD‐11's broader definition (e.g., bullying, stalking, emotional abuse, neglect), and these can be seen in Table 3. Participants indicated on a “Yes” (1) or “No” (0) basis if they had experienced each event in their lifetime such that scores can range from 0 to 21, with higher scores indicating exposure to more traumatic life events. Participants were also asked to identify their most distressing traumatic event and how long ago it occurred.

#### 3.2.2 ICD‐11 PTSD and CPTSD

The ITQ [8] is an 18‐item self‐report measure of all diagnostic requirements for ICD‐11 PTSD and CPTSD. Six items measure the different PTSD and DSO symptoms, respectively, and each symptom cluster (re‐experiencing, avoidance, sense of threat, affective dysregulation, negative self‐concept, and disturbed relationships) is measured by two items. Three items measure functional impairments related to the PTSD and DSO symptoms separately. Participants completed the PTSD items with respect to how bothered they have been by each symptom over the past month and the DSO symptoms in terms of typical reactions. All items use a five‐point Likert scale (0 = *Not at all*, 4 = *Extremely*), and responses of 2 (*Moderately*) or higher indicate that the symptom is present. There is considerable empirical support for the reliability and validity of the ITQ scores [14], and the internal reliability of the PTSD (α = 0.88) and DSO (α = 0.90) scale scores in this sample was acceptable.

#### 3.2.3 Development of the ITQ Clinical Checks

A team of six researchers developed the clinical checks for the ITQ. The team included academic researchers in clinical psychology (MC, TK, CB) and psychological measurement (MS, PH, ER), and most were part of the original ITQ development and validation working group. The development of the clinical checks took place over three phases.

Phase 1 involved an initial meeting to discuss the viability and potential utility of clinical checks in the ITQ. It was agreed that each team member would draft at least one clinical check for each item, and a subsequent meeting would be convened to review, discuss, and agree on the optimal check to be used for each item. To ensure that the final ITQ with clinical checks was still quick and easy to complete and score, it was agreed that there should be one check for each item. Team members were asked to develop the clinical checks based on a set of principles. First, each check should aim to reduce the likelihood of inappropriate endorsements by ensuring that participants responded to the intended clinically relevant meaning, intensity, or duration component of the item. Second, each check should be written in a way that leads to a “Yes” or “No” response; the rationale being that symptoms need to be deemed to be present or absent for diagnostic purposes. Third, the checks should be easily understood. Each member of the team was provided with a copy of the ITQ and a copy of the ICD‐11 description and diagnostic requirements for PTSD (6B40) and CPTSD (6B41) from the ICD‐11 website.

Phase 2 involved another meeting with the aim of selecting a clinical check for each item. The process involved sequentially reviewing and discussing the checks proposed by each team member and selecting one considered to be the best at ensuring the clinical relevance of the symptom was being elucidated in terms of meaning, intensity, or duration. Agreement was reached on selecting a single check for 10 of the 12 ITQ symptom items, while two checks were retained for the outstanding two items due to a lack of consensus as to which item was optimal (these items were those measuring numbing and feeling cutoff from others). It was agreed that the choice of which check to be included in the final version would be based on comparing the responses from the survey data, and the check that produced the largest decrease in item endorsement would be retained. This is in keeping with the overall premise of the clinical checks, which is to reduce the likelihood of false positive responses.

Phase 3 involved the collation of the checks and the production of a draft ITQ with clinical checks (ITQ‐CC) for inclusion in the survey. It was agreed that the clinical checks would only be presented when participants responded to the ITQ items with a score of 2 (*Moderately*) or higher on the Likert scale, which corresponds to the symptom being “present”. During this final meeting, the team discussed the potential of applying a clinical check to the functional impairment items. This was not something that had been pre‐planned, and the idea emerged from discussion. The ITQ items and the associated clinical checks, along with a rationale for each, are presented in Table 2. A copy of the final measure is provided in Appendix 1.

**TABLE 2:** Original ITQ items, clinical checks, and rationale.

ITQ PTSD	Clinical check	Rationale
1. Having upsetting dreams that replay part of the experience or are clearly related to the experience?	Does this happen frequently; at least two times in the last month?	Establishes that nightmares occur regularly enough to be of clinical relevance.
2. Having powerful images or memories that sometimes come into your mind in which you feel the experience is happening again in the here and now?	Do you feel like you are actually reliving the event, even if only for a moment?	Ensures that the “here and now” element of the flashback is present, and endorsement is not just of an intrusive memory.
3. Avoiding internal reminders of the experience (e.g., thoughts, feelings, or physical sensations)?	Do you actively try to push these thoughts out of your mind?	Emphasizes the deliberate and effortfulness nature of the avoidance.
4. Avoiding external reminders of the experience (e.g., people, places, conversations, objects, activities, or situations)?	Have you only started avoiding them since the traumatic experience?	Ensures that the avoidance behaviors are directly related to the traumatic event.
5. Being “super‐alert”, watchful, or on guard?	Do you regularly feel in danger or that something bad is about to happen in certain situations?	Emphasizes the ongoing and pervasive nature of the cognitive‐emotional element of sense of threat.
6. Feeling jumpy or easily startled?	Something normal, like a noise, can shock and set your heart racing – something that doesn't bother other people. Does this happen to you?	Emphasizes physiological reactivity and establishes clinical relevance through comparison with other people's reactions.

ITQ DSO	Clinical check	Rationale
When I am upset, it takes me a long time to calm down.	Do you notice that you get upset more easily than others, *and* have more intense reactions, *and* it takes you longer to calm down compared to other people?	Establishes clinical relevance by ensuring this problem is persistent, pervasive, and more extreme than what is observed in others.
I feel numb or emotionally shut down.	This means being unable to experience emotions such as joy, sadness, excitement, and anger. Is this true for you?	Clarifies meaning and establishes that emotional flattening occurs across a variety of emotions.
	Do you often shut down when you are overwhelmed?	Establishes that this is a frequently occurring problem with emotions.
I feel like a failure.	This does not mean just occasionally feeling bad about yourself. It means consistently viewing yourself as inferior. Is this how you think about yourself?	Emphasizes the ongoing and severe nature of the negative self‐concept. Ensures differentiation for normal and occasional negative thoughts about the self.
I feel worthless.	Some people believe they are unworthy and unimportant. Is this how you feel about yourself?	Ensures accurate understanding of the meaning of this problem.
I feel distant or cut off from people.	This means feeling like other people do not, and cannot, understand you. Is this true for you?	Emphasizes the psychological nature of feeling disconnected from other people in general.
	This means you cannot or do not want to develop strong bonds with other people? Is this true for you?	Emphasizes the behavioral element of feeling disconnected from other people in general.
I find it hard to stay emotionally close to people.	This means fear of conflict or of being rejected if you get close to others. Is this true for you?	Establishes the clinical meaning of this problem which is fear of rejection for other people.

Functional Impairment		
In the past month, have any of these problems affected your relationships or social life? Your work or ability to work? Any other important part of your life such as parenting, or college work, or other important activities?	These questions were about serious and ongoing disruptions in your life; not being able to do the things that you want to do, or things that people normally expect you to do. Do you think that the disruptions are serious and have a negative impact on you?	Emphasizes the severe and pervasive nature of the impact of the problems on functioning.

### 3.3 Data Analysis Plan

Summary statistics for trauma exposure were calculated. Then, we calculated the symptom, symptom cluster, and disorder prevalence estimates with and without the use of the clinical checks. Percentage decreases were calculated using the following formula: (Endorsement% − CC%/Endorsement%) * 100. Statistical comparisons of proportions meeting symptom, symptom cluster, and disorder requirements were made using McNemar's *Z*‐test, which is appropriate for comparing paired‐sample proportions.

## 4 Results

### 4.1 Trauma‐Related Characteristics

In total, 72.5% (*n* = 707) of participants were exposed to at least one traumatic life event, and the mean number of lifetime traumatic events was 3.69 (SD = 4.03; *Mdn* = 3.00). Exposure rates to each traumatic event are displayed in Table 3 and ranged from 3.2% (“caused extreme suffering or death to another person”) to 41.7% (“a loved diagnosed with a life‐threatening illness or experienced a life‐threatening accident”). The events most commonly identified as being most distressing were having a loved one die in an awful manner (14.5%; *n* = 141), learning of a loved one being diagnosed with a life‐threatening illness or involved in a life‐threatening accident (14.3%; *n* = 139), and being repeatedly bullied either online or in person (5.4%; *n* = 53).

**TABLE 3:** Exposure to each traumatic event on the International trauma exposure measure (*N* = 975).

	*n*	%
Diagnosed with a life‐threatening illness.	128	13.1
Someone close to you died in an awful manner.	299	30.7
Someone close to you was diagnosed with a life‐threatening illness or experienced a life‐threatening accident.	407	41.7
Someone threatened your life with a weapon (knife, gun, bomb etc.)	149	15.3
Physically assaulted (punched, kicked, slapped, mugged, robbed etc.) by a parent or guardian.	155	15.9
Physically assaulted (punched, kicked, slapped, mugged, robbed etc.) by someone other than a parent or guardian.	275	28.2
Sexually assaulted (rape, attempted rape, or forced sex acts) by a parent or guardian.	51	5.2
Sexually assaulted (rape, attempted rape, or forced sex acts) by someone other than a parent or guardian.	151	15.5
Sexually harassed (received other types of unwanted sexualized comments or behaviors).	219	22.5
Exposed to war or combat (as a soldier or as a civilian).	49	5.0
Held captive and/or tortured.	37	3.8
You caused extreme suffering or death to another person.	31	3.2
Witnessed another person experiencing extreme suffering or death.	178	18.3
Involved in an accident (e.g., transportation, work, home, leisure) where your life was in danger.	116	11.9
Natural disaster (e.g., hurricane, tsunami, earthquake) where your life was in danger.	59	6.1
Human‐made disaster (e.g., terrorist attack, chemical spill, public shooting) where your life was in danger.	58	5.9
Another person stalked you.	131	13.4
You were repeatedly bullied (online or offline).	294	30.2
You were repeatedly humiliated, put down, or insulted by another person.	291	29.8
You were repeatedly made to feel unloved, unwelcome, or worthless.	298	30.6
You were repeatedly neglected, ignored, rejected, or isolated.	217	22.3

### 4.2 Item Responses and Endorsement Rates

Table 4 presents the ITQ symptom endorsement rates with and without the clinical checks as well as the percentage decreases associated with the use of the clinical checks. Symptom endorsements *without the clinical checks* ranged from 23.9% (feel worthless) to 35.6% (difficulty calming down) whereas endorsements *with the clinical checks* ranged from 15.0% (nightmares) to 26.4% (difficulty calming down). Additionally, the PTSD functional impairment endorsement dropped from 27.4% to 19.7%, and the DSO functional impairment endorsement dropped from 28.9% to 20.9%. Thus, the *percentage decreases* due to the clinical checks ranged from 18.0% (feel like a failure) to 43.9% (hyperalert), and all decreases were statistically significant (*p* < 0.001).

**TABLE 4:** Frequency of ITQ symptom cluster endorsement and clinical checks (CC) (*N* = 975).

	Endorsement		Endorsement + CC		Decreases in cases		McNemar's *Z*
	*N*	%	*N*	%	*N*	%	
PTSD scale							
Nightmares	247	25.3	146	15.0	101	40.7	10.05*
Flashbacks	242	24.8	172	17.6	70	29.0	8.37*
Internal avoidance	281	28.8	230	23.6	51	18.1	7.14*
External avoidance	268	27.5	185	19.0	83	30.9	9.11*
Hyperalert	320	32.8	179	18.4	41	43.9	11.87*
Hyperarousal	243	24.9	182	18.7	61	24.9	7.81*
Functional impairment	267	27.4	192	19.7	75	28.1	8.66*
DSO scale							
Difficulty calming down	347	35.6	257	26.4	90	25.8	9.49*
Numbing	285	29.2	191	19.6^a^	94	32.9^a^	9.70*
			209	21.4^b^	76	26.7^b^	8.72*
Feel like a failure	249	25.5	204	20.9	45	18.0	6.71*
Feel worthless	233	23.9	182	18.7	51	21.8	7.14*
Feel cut off from others	274	28.1	221	22.7^a^	53	19.2^a^	7.28*
			203	20.8^b^	71	26.0^b^	8.43*
Difficult close to others	271	27.8	209	21.4	62	23.0	7.87*
Functional impairment	282	28.9	204	20.9	78	27.7	8.83*

* All *Z*‐values are statistically significant at *p* < 0.001.

^a^ Clinical check 1 for this item.

^b^ Clinical check 2 for this item.

Regarding the two ITQ items for which there were two clinical checks trialed, the percentage decreases were 32.9% and 26.7% for the checks related to the “emotional numbing” symptom, and 19.2% and 26.0% for the checks related to the “feeling cut‐off from others” symptom. Consistent with our pre‐set rule to select the check that led to the largest decrease in symptom endorsement, all subsequent analyzes are based on use of the more stringent checks for these items. Supporting Informations Table 1 includes the finalized ITQ with clinical checks.

Table 5 presents the proportions meeting the symptom cluster requirements with and without the clinical checks. Percentage decreases ranged from 19.1% (Avoidance) to 35.9% (Sense of Threat) and all decreases were statistically significant (*p* < 0.001).

**TABLE 5:** Frequency of ITQ symptom cluster endorsement and clinical checks (CC) (*N* = 975).

	Endorsement		Endorsement + CC		Decrease in cases		McNemar's *Z*
	*N*	%	*N*	%	*N*	%	
Re‐experiencing in the here and now	320	32.8	221	22.7	99	30.8	9.95*
Avoidance	353	36.2	286	29.3	67	19.1	8.19*
Sense of threat	367	37.6	235	24.1	132	35.9	11.49*
PTSD functional impairment	267	27.4	192	19.7	75	28.1	8.66*
Affective dysregulation	418	42.9	318	32.6	100	24.0	10.00*
Negative self‐concept	295	30.3	238	24.4	57	19.5	7.55*
Disturbed relationships	350	35.9	268	27.5	82	23.4	9.06*
DSO functional impairment	282	28.9	204	20.9	78	27.7	8.83*

* All *Z*‐values are statistically significant at *p* < 0.001.

At the disorder level, 5.4% (*n* = 53) met requirements for PTSD without the clinical checks, and this dropped to 3.8% (*n* = 37) with the clinical checks (McNemar's *Z* = 2.17, *p* < 0.001; relative decrease = 29.6%). Moreover, 9.5% (*n* = 93) met requirements for CPTSD without the clinical checks, and this dropped to 4.9% (*n* = 48) with the clinical checks (McNemar's *Z* = 6.56, *p* < 0.001; relative decrease = 48.4%). Consequently, 14.9% (*n* = 146) met diagnostic requirements for either PTSD or CPTSD without the clinical checks, while 8.7% (*n* = 85) met requirements for either disorder with the clinical checks (McNemar's *Z* = 7.68, *p* < 0.001; relative decrease = 41.6%).

## 5 Discussion

The purpose of this study was to describe the process of developing clinical checks for the ITQ and testing their effect in a general population sample. Our results showed that the clinical checks had the intended effects of reducing symptom endorsements and, by extension, estimated rates of disorder prevalence. Reductions in individual symptoms with the clinical checks were quite substantial, ranging from approximately 18%–44%. If one takes seriously the view that the clinical checks operate to remove probable type 1 errors in symptom endorsement, then these findings suggest that such errors are common for all items. One interesting observation was that there was more variability in symptom reductions within the PTSD clusters than within the DSO clusters. As a salient example, the percentage decreases for the two sense‐of‐threat symptoms were 24.9% and 43.9%, whereas the percentage decreases for the two negative self‐concept symptoms were 18.0% and 21.8%. It is difficult to say why this effect occurred, but there are at least two possibilities. One is that some of the PTSD symptom items were more ambiguous than others, and the clinical checks were operating successfully to correct this ambiguity, and the other is that some of the clinical checks for the PTSD items were operating more successfully than others at elucidating the intended clinical meaning, intensity, or duration elements of the symptom item. It will be interesting to monitor future research with the ITQ clinical checks to determine if this is a replicable result.

An important feature of the ITQ is the formal assessment of functional impairment related to both the PTSD and DSO symptoms. It is important to ensure adequate assessment of functional impairment not only because it is a diagnostic requirement for both disorders, but because research shows that while clinicians are primarily interested in assessing symptoms, patients are more focused on the problems caused by the symptoms [15]. Supplementing the functional impairment items in the ITQ with a clinical check led to considerable decreases (approximately 28%) in endorsement for PTSD and DSO‐related impairment. These decreases suggest that the original ITQ functional impairment items may not sufficiently convey the requirement that these are serious and ongoing disruptions to normal daily routines. The importance of considering effective assessment of functional impairment can hardly be understated given its centrality in establishing the presence of disorder. As such, all measures of psychopathology (trauma‐related or otherwise) should include a measure of functional impairment, and perhaps all scale developers should give more consideration as to how to screen for impairment most effectively.

At the diagnostic level, the proportion of people meeting requirements for PTSD or CPTSD dropped by 41.6% (i.e., from 14.9% to 8.7%) when the clinical checks were used. The best estimates of the general population prevalence rate of PTSD are generally considered to come from studies that use large, stratified, random probability‐based samples of the general population and assess PTSD using standardized, structured diagnostic interviews [9]. Studies like this in English‐speaking nations like the UK and the United States find that somewhere between 5% and 8% of the population probably has PTSD. Thus, the ITQ with clinical checks in this study generated a similar overall prevalence rate as would be expected when using structured interview methods. More research will obviously be needed, but this raises the exciting possibility that the addition of simple clinical checks to a standard self‐report measure might reasonably emulate the results of more time‐consuming and costly interview‐based methods of assessment.

One imperfect way of putting the decreases observed in this study into some context is by comparing them to the differences observed between self‐report measures and clinical interviews (however it should be clearly noted that the clinical checks are not meant to emulate a clinical interview). Gelezelyte et al. [16] compared endorsements between the ITQ and the International Trauma Interview [ITI] [17] which is a clinician‐administered diagnostic interview for ICD‐11 PTSD and CPTSD. PTSD symptom endorsements were lower for the ITI, with percentage differences of 22.2% for re‐experiencing, 16.6% for avoidance, and 36.1% for sense of threat, similar to the clinical checks in this study (30.8%, 19.1%, and 35.9%, respectively). Moreover, DSO symptom cluster endorsements were lower for the ITI, with percentage differences of 37.4% for affective dysregulation, 45.7% for negative self‐concept, and 45.6% for disturbed relationships. The impact of the clinical checks for the DSO symptoms was generally smaller (affective dysregulation 24.0%, negative self‐concept 19.5%, disturbed relationships 23.4%). Overall, the percentage decreases observed for the clinical checks are similar to the percentage decreases observed for the ITI relative to the ITQ.

In another study, Kramer et al. [18] compared self‐reported symptoms of DSM‐5 PTSD using the PCL‐5 with those from the CAPS‐5 interview. The authors highlighted what they called “false alarms” which were symptoms considered present based on responses to the PCL‐5 but absent based on CAPS‐5 assessments. This is not perfectly analogous to the “failing” of a clinical check, but it indicates that the initial response to a self‐report item was not supported when additional information was obtained via clinical interview. The ITQ false alarms (calculated as “Endorsement” minus “Endorsement + clinical check”) were lower than those reported by Kramer et al. for the re‐experiencing (Nightmares/Flashbacks: PCL = 8.3%/13.3%, ITQ = 9.8%/7.2%), avoidance (Internal/External avoidance: PCL = 21.7%/33.3%, ITQ = 5.2%/8.5%), and sense of threat (Hyperalert/Hyperarousal: PCL = 18.3%, 25.0%, ITQ = 14.4%, 6.2%) symptoms. The false positives might be lower for the clinical checks than the clinical interview because the interview is more stringent. However, this shows that for all symptoms, across all clusters, there is evidence to suspect that initial responses to self‐report items may generate false positive endorsements (if the CAPS‐5 was considered the correct response), and that follow‐up questions (e.g., clinical checks) can identify these cases.

The generally smaller decreases for the DSO symptoms relative to the PTSD symptoms were, upon reflection, somewhat unexpected. The PTSD items describe experiences that are inherently distressing (e.g., nightmares), are related to the traumatic experience (e.g., avoiding external reminders of the event), or are inherently uncomfortable (e.g., hyperarousal). On the other hand, the DSO items describe feelings and thoughts that are likely to be unpleasant, but not necessarily always distressing and indicative of psychopathology. Experiencing interpersonal disconnection, feelings of low self‐worth, or emotional regulation difficulties, for example, are within the normal range of human experiences and only constitute being symptoms of psychopathology when they are associated with considerable distress and impairment. With the data at hand, there is no way to explain why the clinical checks produced a larger decrease in the PTSD symptoms; maybe the DSO items clearly indicate the clinical aspects of the symptoms and so require less “checking”, or maybe the checks for the DSO symptoms are not as good as those for PTSD at screening out non‐clinical cases. One of the drawbacks of the current study design was that we could not ask people why they said “No” to the clinical check. This will be an important question to address in future studies.

There are some limitations with this study that should be noted. The non‐probability‐based nature of the sample limits the generalizability of the findings to the entire UK population, and it is also unknown whether these results will generalize to clinical samples. Symptom and endorsement rates are likely to be considerably higher within clinical populations, so it will be important to determine what effect the clinical checks have in this context. Additionally, we could not assess any of the participants with a clinical interview to evaluate concordance between interview assessments and the ITQ with clinical checks. This will be an interesting area for study, but as we have already noted, the clinical checks are not intended to emulate a clinical interview. Finally, we do not know anything about who failed to pass the clinical checks and why. This will be a key focus of future research.

In conclusion, this study provides initial evidence of the effect of using clinical checks within a popular measure of ICD‐11 PTSD and CPTSD. For the longest time, researchers and clinicians interested in assessing psychiatric disorders have had a choice between self‐report questionnaires and clinician‐administered diagnostic interviews. These methods have their respective strengths and weaknesses [19], but we believe the approach described in this study—the addition of clinical checks within self‐report measures—offers another option that captures many of the greatest strengths of these existing approaches.

## Conflicts of Interest

The authors declare no conflicts of interest.

### Peer Review

The peer review history for this article is available at https://www.webofscience.com/api/gateway/wos/peer‐review/10.1111/acps.13799.

## Supporting information


**Table S1.** Original ITQ items, clinical checks, and rationale.

## Data Availability

Research data are not shared.

## Appendix 1 The International Trauma Questionnaire With Clinical Checks

**Instructions:** Please answer the following questions thinking about the traumatic event you previously identified as the most distressing [respondents should have been screened for trauma prior to the administration of this scale]. Below are several problems that people sometimes report in response to traumatic or stressful life events. Please read each item carefully, then circle one of the numbers to the right to indicate how much you have been bothered by that problem in the past month.

**Table:**

	Not at all	A little bit	Moderately	Quite a bit	Extremely
P1. Having upsetting dreams that replay part of the experience or are clearly related to the experience?	0	1	2	3	4
If scored 2 or higher, please answer: Does this happen frequently; at least two times in the last month?			YesNo		
P2. Having powerful images or memories that sometimes come into your mind in which you feel the experience is happening again in the here and now?	0	1	2	3	4
If scored 2 or higher, please answer: Do you feel like you are actually reliving the event, even if only for a moment?			Yes No		
P3. Avoiding internal reminders of the experience (for example, thoughts, feelings, or physical sensations)?	0	1	2	3	4
If scored 2 or higher, please answer: Do you actively try to push these thoughts out of your mind?			Yes No		
P4. Avoiding external reminders of the experience (for example, people, places, conversations, objects, activities, or situations)?	0	1	2	3	4
If scored 2 or higher, please answer: Have you only started avoiding them since the traumatic experience?			Yes No		
P5. Being “super‐alert”, watchful, or on guard?	0	1	2	3	4
If scored 2 or higher, please answer: Do you regularly feel in danger or that something bad is about to happen in certain situations?			Yes No		
P6. Feeling jumpy or easily startled?	0	1	2	3	4
If scored 2 or higher, please answer: Something normal, like a noise, can shock and set your heart racing – something that doesn't bother other people. Does this happen to you?			Yes No		
In the past month have the above problems					
P7. Affected your relationships or social life?	0	1	2	3	4
P8. Affected your work or ability to work?	0	1	2	3	4
P9. Affected any other important part of your life such as parenting, or school or college work, or other important activities?	0	1	2	3	4
If P7, P8 or P9 scored 2 or higher, please answer: These questions were about serious and ongoing disruptions in your life; not being able to do the things that you want to do, or things that people normally expect you to do. Do you think that the disruptions are serious and have a negative impact on you?			Yes No		

**Instructions:** Below are problems that people who have had stressful or traumatic events sometimes experience. The questions refer to ways you typically feel, ways you typically think about yourself, and ways you typically relate to others. Answer the following thinking about how true each statement is of you.

**Table:**

How true is this of you?	Not at all	A little bit	Moderately	Quite a bit	Extremely
C1. When I am upset, it takes me a long time to calm down.	0	1	2	3	4
If scored 2 or higher, please answer: Do you notice that you get upset more easily than others, *and* have more intense reactions, *and* it takes you longer to calm down compared to other people?			Yes No		
C2. I feel numb or emotionally shut down.	0	1	2	3	4
If scored 2 or higher, please answer: This means being unable to experience emotions such as joy, sadness, excitement, and anger. Is this true for you?			Yes No		
C3. I feel like a failure.	0	1	2	3	4
If scored 2 or higher, please answer: This does not mean just occasionally feeling bad about yourself. It means consistently viewing yourself as inferior. Is this how you think about yourself?			Yes No		
C4. I feel worthless.	0	1	2	3	4
If scored 2 or higher, please answer: Some people believe they are unworthy and unimportant. Is this how you feel about yourself?			Yes No		
C5. I feel distant or cut off from people.	0	1	2	3	4
If scored 2 or higher, please answer: This means you cannot or do not want to develop strong bonds with other people? Is this true for you?			Yes No		
C6. I find it hard to stay emotionally close to people.	0	1	2	3	4
If scored 2 or higher, please answer: The means fear of conflict or of being rejected if you get close to others. Is this true for you?			Yes No		
In the past month, have the above problems in emotions, in beliefs about yourself and in relationships					
C7. Created concern or distress about your relationships or social life?	0	1	2	3	4
C8. Affected your work or ability to work?	0	1	2	3	4
C9. Affected any other important parts of your life such as parenting, or school or college work, or other important activities?	0	1	2	3	4
If C7, C8 or C9 2 or higher, please answer: These questions were about serious and ongoing disruptions in your life; not being able to do the things that you want to do, or things that people normally expect you to do. Do you think that the disruptions are serious and have a negative impact on you?			Yes No		
