# Supplementary material for: Testing the Use of “Clinical Checks” With the International Trauma Questionnaire to Measure PTSD and Complex PTSD
Source: Acta Psychiatr Scand. 2025 Mar 23;152(1):49–59. doi: 10.1111/acps.13799 (PMC12127064; doi:10.1111/acps.13799)
Supplement: Supplementary file 1 — Table S1. Original ITQ items, clinical checks, and rationale. [file ACPS-152-49-s001.docx]

**Supplementary Table 1**

*Original ITQ items, clinical checks, and rationale.*

| **ITQ PTSD** | Clinical check | Rationale |
| --- | --- | --- |
| 1. Having upsetting dreams that replay part of the experience or are clearly related to the experience? | Does this happen frequently; at least two times in the last month? | Establishes that nightmares occur regularly enough to be of clinical relevance. |
| 2. Having powerful images or memories that sometimes come into your mind in which you feel the experience is happening again in the here and now? | Do you feel like you are actually reliving the event, even if only for a moment? | Ensures that the ‘here and now’ element of the flashback is present, and endorsement is not just of an intrusive memory |
| 3. Avoiding internal reminders of the experience (for example, thoughts, feelings, or physical sensations)? | Do you actively try to push these thoughts out of your mind? | Emphasises the deliberate and effortfulness nature of the avoidance. |
| 4. Avoiding external reminders of the experience (for example, people, places, conversations, objects, activities, or situations)? | Have you only started avoiding them since the traumatic experience? | Ensures that the avoidance behaviours are directly related to the traumatic event. |
| 5. Being “super-alert”, watchful, or on guard? | Do you regularly feel in danger or that something bad is about to happen in certain situations? | Emphasises the ongoing and pervasive nature of the cognitive-emotional element of the sense of threat. |
| 6. Feeling jumpy or easily startled? | Something normal, like a noise, can shock and set your heart racing – something that doesn’t bother other people. Does this happen to you? | Emphasises the physiological reactivity component of this problem and establishes clinical relevance through comparison with other people’s reactions. |
| **ITQ DSO** | **Clinical check** | **Rationale** |
| When I am upset, it takes me a long time to calm down. | Do you notice that you get upset more easily than others, *and* have more intense reactions, *and* it takes you longer to calm down compared to other people? | Establishes clinical relevance by ensuring this problem is persistent, pervasive, and more extreme than what is observed in others. |
| I feel numb or emotionally shut down. | This means being unable to experience emotions such as joy, sadness, excitement, and anger. Is this true for you? | Clarifies the meaning of the problem and establishes that emotional flattening occurs across a variety of emotions. |
| I feel like a failure. | This does not mean just occasionally feeling bad about yourself. It means consistently viewing yourself as inferior. Is this how you think about yourself? | Emphasises the ongoing and severe nature of the negative self-concept. Ensures differentiation for normal and occasional negative thoughts about the self. |
| I feel worthless. | Some people believe they are unworthy and unimportant. Is this how you feel about yourself? | Ensures accurate understanding of the meaning of this problem. |
| I feel distant or cut off from people. | This means you cannot or do not want to develop strong bonds with other people? Is this true for you? | Emphasises the behavioral element and enduring nature of feeling disconnected from other people in general. |
| I find it hard to stay emotionally close to people. | This means fear of conflict or of being rejected if you get close to others. Is this true for you? | Establishes the clinical meaning of this problem which is fear of rejection for other people. |
| **Functional Impairment** |  |  |
| In the past month, have any of these problems affected your relationships or social life? Your work or ability to work? Any other important part of your life such as parenting, or college work, or other important activities? | These questions were about serious and ongoing disruptions in your life; not being able to do the things that you want to do, or things that people normally expect you to do. Do you think that the disruptions are serious and have a negative impact on you? | Emphasizes the severe and pervasive nature of the impact of the problems on functioning. |
